# Advances and Applications of Plant Base Editing Technologies

**DOI:** 10.3390/ijms26199452

**Published:** 2025-09-27

**Authors:** Hao Peng, Jiajun Li, Kehui Sun, Huali Tang, Weihong Huang, Xi Li, Surong Wang, Ke Ding, Zhiyang Han, Zhikun Li, Le Xu, Ke Wang

**Affiliations:** 1 MARA Key Laboratory of Sustainable Crop Production in the Middle Reaches of the Yangtze River (Co-Construction by Ministry and Province), Hubei Key Laboratory of Waterlogging Disaster and Agricultural Use of Wetland, College of Agriculture, Yangtze University, Jingzhou 434025, China; ph17723104003@163.com; 2 State Key Laboratory of Crop Gene Resources and Breeding, National Key Facility for Crop Gene Resources and Genetic Improvement, Institute of Crop Sciences, Chinese Academy of Agricultural Sciences, Beijing 100081, China; 15028361534@163.com (J.L.); sunkehui200102@163.com (K.S.); tanghuali@caas.cn (H.T.); hwh17669495484@163.com (W.H.); lixi3925@163.com (X.L.); wangsurong@stu.sicau.edu.cn (S.W.); dingk0916@163.com (K.D.); hzy1787324918@163.com (Z.H.); 3 State Key Laboratory of North China Crop Improvement and Regulation, North China Key Laboratory for Crop Germplasm Resources of Education Ministry, Hebei Agricultural University, Baoding 071001, China; lzhk@hebau.edu.cn

**Keywords:** base editing, CRISPR/Cas9, crop improvement, molecular breeding, genome engineering

## Abstract

Base editing represents a major breakthrough in the field of genome editing in recent years. By fusing deaminases with the CRISPR/Cas system, it enables precise single-base modifications of DNA. This review systematically summarizes the development of base editing technologies, including cytosine base editors (CBEs), adenine base editors (ABEs), and glycosylase base editors (GBEs), with a particular focus on their applications in crop improvement as well as future trends and prospects. We highlight advances in the creation of novel germplasm with enhanced stress resistance and desirable agronomic traits through base editing in rice, wheat, maize, potato, and other crops, particularly for improving herbicide resistance, disease resistance, and grain quality. Furthermore, we analyze factors that influence base editing efficiency, noting that challenges remain, such as PAM sequence constraints, limited base conversion types, off-target effects, narrow editing windows, and efficiency variation. Future efforts should aim to optimize deaminase activity, expand PAM compatibility, and develop versatile tools to facilitate the broad application of base editing in molecular breeding. This review provides a timely reference for researchers and breeders, offering theoretical guidance and practical insights into harnessing base editing for crop genetic improvement.

## 1. Introduction

Gene editing technology, as a molecular tool capable of precisely modifying specific sequences of the genome in living organisms, has been widely applied since its emergence in plant and animal gene function analysis, disease treatment research, and genetic improvement, becoming a core driving force for the development of life sciences. The development of this technology has undergone three major innovations: zinc finger nuclease (ZFN) technology [1] achieved targeted gene editing for the first time; transcription activator-like effector nuclease (TALEN) technology [2] significantly improved flexibility of target recognition through modular design; and the CRISPR/Cas system [3], developed based on the bacterial immune mechanism, has become the mainstream tool due to its high efficiency and low cost. The core principle of these technologies relies on the synergistic action of engineered nucleases and specific recognition elements, inducing double-strand breaks (DSBs) at genomic target sites, which trigger repair by non-homologous end joining (NHEJ) or homologous recombination (HR) [4,5], during which repair errors lead to target site mutations. However, the early gene editing technologies could only cause gene silencing: the editing process was accompanied by random insertions/deletions (indels), making it difficult to achieve precise replacement at the base level. Notably, many desirable agronomic traits in crops (such as stress resistance and yield composition) are often closely associated with point mutations or single nucleotide polymorphisms (SNPs) [6,7]. For example, a base variation in the rice *OsSPL14* gene can significantly increase yield [8], and a specific SNP in the wheat *TaGW2* gene can enlarge grain size [9]. A base change in a gene can generate superior traits in plants and accelerate crop improvement. Therefore, developing base editing technology that enables precise and efficient base mutations in crops is of great importance and has enormous application prospects in crop breeding research. Against this background, base editing (BE) technology based on the CRISPR/Cas9 system emerged. Since the development of the first-generation base editor, various laboratories have adopted multiple strategies to optimize it, aiming to improve editing efficiency and broaden its range of applications. At present, base editing technology has been successfully applied in multiple crops such as rice, wheat, and maize, for the purpose of improving agronomic traits and enhancing stress resistance. This article systematically analyzes the molecular mechanisms and optimization pathways of base editing technologies, reviews innovative application cases in major staple crops, and discusses its developmental potential and challenges in molecular design breeding.

## 2. Technical Advances

The core working mechanism of base editing technology relies on a complex system composed of a deaminase, SpCas9 variants, and sgRNA [10]. Guided by the sgRNA, this complex specifically recognizes the target DNA sequence, while the deaminase catalyzes the deamination reaction to achieve precise base substitution. The earliest SpCas9 variant used was dCas9, which harbors double mutations (Asp10Ala and His840Ala) that abolish nuclease activity while retaining DNA-binding ability. The Cas9 variant most commonly used today is nCas9, which carries a single Asp10Ala mutation that preserves only single-stranded DNA nicking activity [10]. Depending on the type of deaminase employed and the category of base conversion, currently developed base editing systems can be broadly divided into three classes: cytosine base editors (CBEs), adenine base editors (ABEs), and glycosylase base editors (GBEs).

### 2.1. Cytosine Base Editing System (CBE)

**Figure 1:**
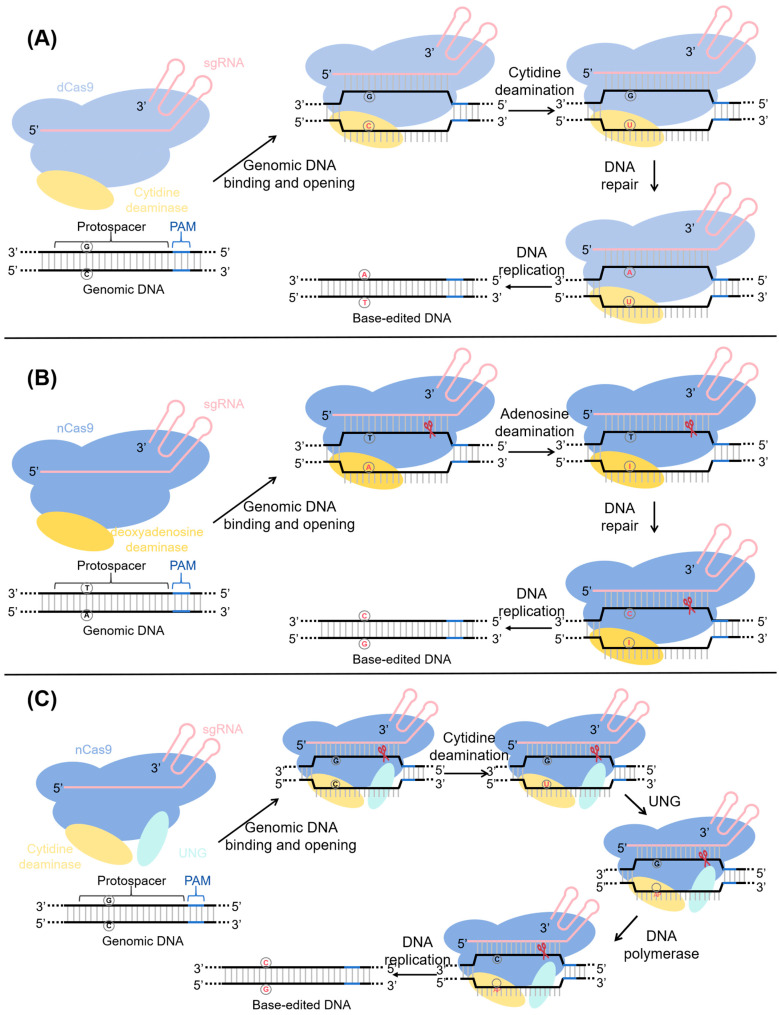
(**A**) Schematic diagram of the cytosine base editing system (CBE) editing mechanism; (**B**) Schematic diagram of the adenine base editing system (ABE) editing mechanism; (**C**) Schematic diagram of the glycosylas base editing system (GBE) editing mechanism.

The first-generation cytosine base editing system (CBE) was successfully developed by David R. Liu’s group in 2016 based on the CRISPR/Cas9 platform, consisting of a fusion protein of dCas9 and a cytidine deaminase together with an sgRNA. Four different cytidine deaminases—hAID, hAPOBEC3G, rAPOBEC1, and pmCDA1—were evaluated for their deamination activity on single-stranded DNA, and rAPOBEC1 (rAC1) from rat showed the highest activity. This enzyme was fused via an amino acid linker to the N-terminus of dCas9 to generate CBE1, but its C-to-T editing efficiency in HEK293T cells was only 0.8–7.7% [10]. The editing process of this system (Figure 1A) involves three key steps: sgRNA directs the fusion protein to the target DNA sequence, the cytidine deaminase catalyzes the deamination of cytosine (C) to uracil (U), and subsequently U is recognized as thymine (T) during DNA replication or repair, while the complementary guanine (G) is replaced by adenine (A), thereby achieving precise C/G-to-T/A base substitution. To improve efficiency, successive optimization of CBE was carried out. Komor et al. [10] first developed CBE2 by fusing a uracil DNA glycosylase inhibitor (UGI) to the C-terminus of CBE1, which blocked uracil excision repair, increased efficiency threefold to 20%, and reduced unintended indels. They then replaced dCas9 with nCas9, which retains single-strand nicking activity, generating CBE3 and further improving editing efficiency by 2–6 fold, up to 37%. Subsequently, researchers developed CBE4 by adding a second UGI to the C-terminus of CBE3 and extending the linker sequences between the deaminase, nCas9, and UGI. This new editor achieved 15–90% efficiency, representing a 50% improvement over CBE3 [11]. Later studies demonstrated that nuclear localization signals (NLSs) also influenced base editing performance. Koblan et al. [12] tested six NLS configurations and found that adding a bipartite NLS (bpNLS) to both the N- and C-termini of CBE4 yielded the best performance. With further codon optimization of the entire construct, including APOBEC1 and nCas9 domains, they created CBE4max, which enhanced editing efficiency across various cell types by 1.8–9 fold, reaching up to 89%. Particularly at low dosage or at difficult-to-edit sites, CBE4max showed remarkable advantages, for example, achieving 77% efficiency at the SCN9a splice acceptor site in mouse neuroblastoma cells, a 5.5-fold increase compared to CBE4 (14%). Whether optimizing codons or nuclear localization signals (NLS), the ultimate goal is to enhance editing efficiency by increasing the expression level of nCas proteins in the nucleus. Given the critical role of cytidine deaminases in base editing, extensive efforts have been made to identify or engineer novel deaminases with improved properties. Gehrke et al. (2018) [13] replaced rAPOBEC1 with human APOBEC3A (A3A) and introduced an N57G mutation to create eA3A, which conferred higher precision by favoring TC motifs, though with an efficiency of 20–50% similar to CBE3. Thuronyi et al. (2019) [14] employed the bacteriophage-assisted continuous evolution (BE-PACE) system to evolve three deaminases: APOBEC1, CDA1, and FERNY (the latter reconstructed through ancestral sequence reconstruction from node 656 of the APOBEC family phylogenetic tree, which is 29% smaller than APOBEC1). This process yielded evoAPOBEC1 (H122L+D124N), evoCDA1 (A123V), and evoFERNY (H102P+D104N). These variants were subsequently incorporated into the CBE4max editor. EvoAPOBEC1-BE4max exhibited markedly enhanced editing efficiency at GC-rich sites, increasing from 2.3% to 58% at the HEK3 locus. EvoFERNY-BE4max showed even higher activity at GC-rich sites than evoAPOBEC1, achieving 70% efficiency at the HEK3 locus. In contrast, evoCDA1-BE4max significantly improved editing at difficult-to-target loci, such as the TMC1 hearing-loss model, reaching 24% efficiency, which represents a 2.6-fold increase over CDA1-BE4max. Interestingly, Chen et al. [15] engineered an adenine deaminase TadA-8e by introducing an N46L mutation to abolish its adenine deaminase activity and confer cytidine deamination capability, while Neugebauer et al. [16] further evolved TadA-8e using phage-assisted approaches to generate TadA-CD variants with strong activity toward deoxycytidine. These engineered TadA-8e variants were used to develop new CBEs (Td-CBE or TadCBE). In HEK293T cells, Td-CBEmax (E27R) achieved C-to-T editing efficiencies of 57.7–94.9% at 13 endogenous loci, compared with 44.9–93.2% for BE4max [15], while TadCBE averaged 51–60% efficiency across nine sites, comparable to BE4max (47%) and evoAPOBEC1-BE4max (55%), but superior to evoFERNY-BE4max (41%) [16]. Notably, TadA-derived CBEs not only maintained or exceeded the editing activity of conventional CBEs but also offered smaller size, lower indel frequencies, and dramatically reduced Cas-independent DNA and RNA off-target editing.

### 2.2. Adenine Base Editing System (ABE)

Following the development of the cytosine base editing system (CBE), the team led by David R. Liu successfully established the adenine base editing system (ABE) in 2017, with the key innovation being the replacement of the cytosine deaminase in CBE with an engineered adenine deaminase. By fusing a mutated TadA* (TadA A106V and D108N) with nCas9, the researchers constructed the first-generation adenine base editor, ABE1.2, which achieved an editing efficiency of only 3.2 ± 0.88% [17]. The working principle of ABE is similar to that of CBE (Figure 1B): sgRNA directs the nCas9–deaminase fusion protein to a specific DNA site, where the adenine deaminase catalyzes the deamination of adenine (A) to produce inosine (I). During DNA replication, I is recognized as guanine (G), while the complementary thymine (T) is corrected to cytosine (C), thereby achieving a targeted A/T-to-G/C base pair conversion. Importantly, naturally occurring adenosine and adenine deaminases such as *E. coli* TadA and ecTadA, human ADAR2, and mouse ADA act exclusively on various forms of RNA. Through directed evolution, the research team re-engineered TadA and successfully endowed it with DNA editing activity, representing a critical breakthrough in the realization of ABE technology [17]. To further enhance editing efficiency, Gaudelli and colleagues [17] subjected wild-type TadA to artificial evolution using error-prone PCR, DNA shuffling, and other strategies to generate random amino acid substitutions, and after seven rounds of optimization, developed a series of ABE systems ranging from ABE1.2 to ABE7.10. ABE7.10 was designed by fusing nCas9 with a heterodimer of wild-type TadA and an evolved TadA7.10, which increased the editing efficiency from 3% to 58%. Koblan et al. [12] replaced the SV40 NLS of ABE7.10 with a bipartite nuclear localization signal (bis-bpNLS) and optimized codon usage with a GenScript strategy, generating ABEmax, which further improved efficiency, achieving 46% editing at the HBG promoter site—seven times higher than ABE7.10. Gaudelli’s team [18] next evolved ABE7.10 using an adenine deaminase variant library to create the ABE8 series (40 variants classified into ABE8.x-m, monomers composed solely of the evolved TadA* without wild-type TadA, about 500 bp shorter in sequence than dimeric versions, and ABE8.x-d, heterodimers fusing wild-type TadA with evolved TadA*). Among these, ABE8.20-m showed optimal performance, reaching 60% editing efficiency in CD34 cells and as high as 98–99% in primary human T cells, thereby greatly expanding editing efficiency and applicability. Meanwhile, Richter et al. [19] applied phage-assisted non-continuous and continuous evolution (PANCE and PACE) to evolve the deaminase component of ABE7.10, producing ABE8e, which exhibited deamination activity 590 times higher than ABE7.10, with editing efficiencies in mammalian cells increasing from 1.7–20% to 18–86%.

### 2.3. The Glycosylase Base Editor (GBE)

The glycosylase base editor (GBE) was first developed by Zhao et al. in 2020 [20], overcoming the limitation of cytosine base editors (CBEs) and adenine base editors (ABEs), which could only mediate transitions between pyrimidines or between purines, and enabling pyrimidine-to-purine conversions. GBE1.0 achieved editing efficiencies ranging from 5.3% to 53.0% in mammalian cells. The core components of GBE include a cytidine deaminase, uracil-DNA glycosylase (UNG), nCas9, and sgRNA. Guided by the sgRNA, the fusion protein binds to the target DNA, where the cytidine deaminase converts cytosine (C) into uracil (U). UNG subsequently removes U, generating an apurinic/apyrimidinic (AP) site. The formation of this AP site, together with a nick introduced by nCas9 on the non-edited strand, triggers DNA repair and replication, thereby favoring the insertion of guanine (G) at the AP site and ultimately achieving precise C/G-to-G/C base pair conversion (Figure 1C). In the same year, Kurt and colleagues [21] developed the CGBE1 and miniCGBE1 editors for C/G-to-G/C transversions. CGBE1 consists of an RNA-guided Cas9 nickase, an *Escherichia coli*–derived uracil DNA N-glycosylase (eUNG), and a rat APOBEC1 cytidine deaminase variant (R33A), achieving an average editing efficiency of 14.4% across 25 sites. MiniCGBE1, generated by removing the eUNG domain, showed a slightly reduced average efficiency of 13% but significantly lowered the frequency of insertions and deletions (indels). To further enhance editing efficiency, Sun et al. [22] subsequently developed GBE2.0 (APOBEC(R33A)-nCas9-Rad51-Ung1), which incorporated several key enhancements. These included replacing human Ung with the more active yeast Ung1, introducing the APOBEC1 R33A variant, and fusing the DNA repair protein Rad51. The single-stranded DNA-binding domain (ssDBD) of Rad51 plays a critical role in DNA repair and enhances the deaminase’s affinity for ssDNA, thereby boosting the editor’s efficiency. This optimized version achieved an average editing efficiency of 30.88% across 17 chromosomal loci, representing a twofold increase compared with GBE1.0 (15.54%). Dong et al. [23] adopted an activation-based strategy by fusing VP64 to GBE to generate VP64-GBE, which significantly improved performance, yielding an average efficiency of 24.96% across 18 sites in HEK293T cells, compared with only 11.91% for the original GBE. Further incorporation of the SunTag system produced SunTag-GBEs, which not only enhanced efficiency and product purity but also expanded the effective editing window to the C6–C8 positions, raising the editing efficiency at the hard-to-edit locus PPP1R12C-5 to 35%. Chen et al. [24] developed another class of CGBEs by harnessing the base excision repair (BER) pathway. Based on CBE3 and CBE4, they removed UGI and fused core BER proteins including XRCC1, DNA ligase III (LIG3), and the 8-kDa domain of DNA polymerase β (PB), constructing more than 30 editors. Among them, rAPOBEC-nCas9-rXRCC1 performed best, achieving an average efficiency of 15.4% for C-to-G editing in human cells, with a maximum of 37%. Interestingly, by introducing an N46L mutation into TadA-8e to abolish its adenine deaminase activity, researchers generated a TadA-8e derivative with cytidine deaminase activity. The resulting Td-CGBE enabled highly efficient and precise C/G-to-G/C editing, reaching up to 72.8% efficiency in HEK293T cells [15].

## 3. Application of Base Editing Technologies in Plants

Base editing technology represents a breakthrough advancement in the field of genome editing, with its core advantage lying in the ability to achieve precise base substitution at target sites without inducing DNA double-strand breaks (DSBs). Since its emergence, this technology has rapidly attracted widespread attention within the scientific community and has demonstrated enormous potential in studies involving animals, plants, and microorganisms. By enabling the precise manipulation of individual nucleotide substitutions in target genes, it can generate both loss-of-function mutations and gain-of-function variations, thereby providing a revolutionary tool for crop gene functional analysis, genetic improvement, de novo domestication, and directed evolution. Beginning with the third-generation cytosine base editor (CBE) and adenine base editor (ABE), researchers have continuously refined and expanded applications of base editors in plants. Strategies such as codon optimization and deaminase engineering have been employed to significantly improve editing efficiency, and the technology has now been successfully applied in staple crops such as rice and wheat, as well as horticultural crops like potato and tomato.

### 3.1. Optimization of Base Editing Technologies in Plants

Zong et al. (2017) [25] constructed a plant base editor (nCas9-PBE) by combining rat APOBEC1 cytidine deaminase, nCas9, and the uracil glycosylase inhibitor (UGI), then they performed codon optimization for cereal plants and cloning under the maize ubiquitin-1 (Ubi-1) promoter. In rice, wheat, and maize protoplasts, the system achieved C-to-T editing efficiencies of 5.8%, 6.8%, and 4.0%, respectively. At the *OsSPL14* locus in rice, the base C-to-T editing efficiency reached 7.07%, while at the *TaLOX2* locus in wheat, multiplexed C-to-T editing reached 12.48%. Building on this system, Zong et al. (2018) [26] replaced rat APOBEC1 with codon-optimized human APOBEC3A for cereals, generating A3A-PBE. This editor reached C-to-T editing efficiencies of 24.5% in rice protoplasts and up to 82.9% in regenerated rice plants at the *OsCDC48* locus. In wheat protoplasts, four target sites showed an average efficiency of 13.1%, and in regenerated plants, the *TaALS* locus reached 22.5%. Notably, the editing window of A3A-PBE expanded to 17 nucleotides (positions 1–17), compared with only 7 nucleotides (positions 3–9) for the original PBE. Zeng et al. (2020) [27] codon-optimized hA3A, evoAPOBEC1, evoCDA1, and evoFERNY for rice and paired them with rice codon-optimized Cas9n-NG [28], which recognizes NG PAM sites, to construct four PhieCBEs: PevoAC1-NG, PevoFERNY-NG, PevoCDA1-NG, and PhA3A-max-NG. Among them, PevoFERNY-NG demonstrated the most robust overall editing characteristics, achieving C-to-T efficiencies as high as 86.3% in rice. Li et al. (2018) [29], based on the ABE7.10 architecture (ecTadA-ecTadA* fused to nCas9-D10A), constructed seven plant ABE variants (PABE-1 to PABE-7) by altering the position of the deaminase and modifying the number and placement of nuclear localization signals (NLSs). PABE-7 (deaminase at the N-terminus with three C-terminal NLSs) performed best, reaching 32.8% A-to-G efficiency in rice and wheat protoplasts, 15.8–59.1% in regenerated rice plants, but only 0.4% and 1.1% in regenerated wheat plants.Yan et al. (2018) [30] optimized codons of wild-type E. coli TadA and its mutant TadA*7.10 and fused them to nCas9 to construct rBE14, which enabled A-to-G editing in rice genes *OsMPK6*, *OsWRKY13*, *OsSERK2*, and *OsWRKY45*, with efficiency peaking at 62.26% in *OsWRKY45*. Wei et al. (2021) [31] constructed rABE8e using codon-optimized ABE8e for plants, achieving 85.7% and 82.1% A-to-G editing efficiencies at EPSPS and ALS loci in rice, with some *Wx* gene targets reaching 100%. Yan et al. (2021) [32] introduced V82S/Q154R mutations into TadA8e to generate TadA9, and constructed rBE46b and rBE48b using TadA9 and TadA8e, respectively. In rice, rBE46b achieved A-to-G efficiencies of 56.25%, 86.96%, and 97.92% at *OsMPK6*, *OsMPK13*, and *Tms9-1*, while rBE48b efficiencies were 58.33%, 18.75%, and 14.29%. Interestingly, TadA9 monomers displayed comparable or even superior activity within the editing window.Tan et al. (2022) [33] developed hyper ABE8e (hyABE8e) by inserting the single-stranded DNA-binding domain (DBD) from RADIATION SENSITIVE 51 (Rad51) [34] between TadA8e and nCas9 and performing codon optimization for rice. This system reached an average A-to-G editing efficiency of 78.5% across 29 rice loci, with four sites (TS4, TS12, TS14, TS16) achieving 100% efficiency. Sretenovic et al. (2021) [35] adapted CGBE for plants by using maize codon-optimized Cas9 (zCas9) and rice codon-optimized components, producing three versions: pYPQ265K (UNG-rAPOBEC1(R33A)-zCas9(D10A)), pYPQ265N1 (rAPOBEC1-zCas9(D10A)-UNG), and pYPQ265O1 (rAPOBEC1-zCas9(D10A)-rXRCC1). In rice and tomato protoplasts, pYPQ265K showed the highest efficiency, achieving C-to-G editing rates of 0.25–1.75% and 0.3–0.7%, respectively. In rice T_0_ plants, only pYPQ265O1 generated C-to-G edits at the OsALS-sgRNA32 locus, with an efficiency of 4.8%, though all versions produced C-to-T byproducts. Tian et al. (2022) [36] improved UNG codon optimization and used highly active deaminases to construct OsCGBE03, which achieved only 1.69% efficiency in rice protoplasts but reached an average of 21.3% C-to-G efficiency across five genes (*OsIPA1*, *OsbZIP5*, *OsSIR1*, *OsALS1*, *NRT1.18*) in rice plants. Fan et al. (2024) [37] tested TadA-8e-derived cytosine base editors (TadCBEa, TadCBEd, TadCBEd V106W) in rice and tomato protoplasts, achieving high-efficiency, high-purity, and low-off-target C-to-T editing. In rice protoplasts, efficiency peaked at 55.4%, with extremely low indel rates, making these editors superior to traditional CBEs for applications requiring minimal off-target effects and byproducts. Most recently, Jiang et al. [38] replaced human UNG (hUNG) in CGBEs with cod UNG (coUNG), a uracil DNA glycosylase derived from cold-adapted codfish *Gadus morhua*, thereby improving C-to-G editing efficiency by 1.71- to 2.54-fold. In rice T_0_ plants, efficiencies ranged from 33.33% to 68.75%. They further integrated TadA-8e-derived cytidine deaminase components (TadA-CDs) by fusing coUNG with TadA-CDc and SpCas9 nickase, generating CDc-CGBEco. This editor achieved homozygous C-to-G editing without byproducts in up to 52.08% of transgenic lines, while heterozygous editing reached 83.33%, with no significant off-target effects observed.

### 3.2. Application of Base Editing Technology in Rice and Wheat

With the ongoing optimization of plant base editors in terms of efficiency, specificity, and editing window, their applications have rapidly expanded from fundamental research to practical crop improvement. A series of highly efficient and precise editing systems have established a solid technical foundation for the targeted development of novel germplasm in major crops, demonstrating promising potential in rice, wheat, and a variety of other plant species.

As one of the world’s most important staple crops, rice was the first system in which base editing technology was tested and successfully applied. As shown in Table 1, base editing was applied to the relevant rice genes to confer important agronomic traits, including herbicide resistance and improved nitrogen use efficiency. In 2017, Li et al. [39] used BE3 to target three sites within the *OsSBEIIb* gene, which encodes starch branching enzyme IIb, and the *OsPDS* gene, which encodes phytoene desaturase. Their work demonstrated that base editing achieved much higher efficiency in targeted base correction compared with HDR-mediated gene replacement, while also showing that base-editing vectors were easier to construct than HDR plasmids (Table 1). In the same year, Lu and Zhu [40] employed a base editor lacking the UGI domain to edit the *NRT1.1B* gene, which encodes a nitrogen transporter, and the *SLR1* gene, which encodes a DELLA protein where amino acid substitutions in or near its TVHYNP motif reduce plant height. They achieved C-to-T/G editing efficiencies of 2.7% and 13.3%, respectively (Table 1). In 2018, Li et al. [29] introduced point mutations into the acetyl-CoA carboxylase (*ACC*) gene of rice using base editing, thereby generating herbicide-resistant rice lines (Table 1). In 2020, Kuang et al. [41] employed both CBE and ABE to generate nearly saturated mutations of the endogenous herbicide target gene *OsALS1*, uncovering novel herbicide-resistance loci and creating new resistant germplasm (Table 1). In the same year, Liu et al. [42] performed precise base editing of the α-tubulin homologous gene *OsTubA2*, producing rice germplasm resistant to the herbicides trifluralin and dinitroaniline (Table 1). In 2022, Tian et al. [36] used the newly developed OsCGBE03 base editor to target genes such as *OsIPA1*, *OsbZIP5*, *OsSIR1*, and *OsALS1*, successfully conferring either high nitrogen-use efficiency or herbicide resistance to rice plants (Table 1).

Although many studies have demonstrated successful applications of base editing in rice, reports in wheat remain comparatively scarce. In 2019, Zhang et al. [43] applied ABE to edit the *TaALS-P174* gene, which encodes acetolactate synthase, generating herbicide-resistant wheat. Double mutants of *TaALS-P174* and *TaALS-G631* showed slightly greater resistance to pyroxsulam than single mutants, while *ALS* mutations in *TaALS-P174* conferred strong resistance to this herbicide. Using CBE, they also introduced a substitution at position 174 of the *TaALS* gene, converting proline to serine, alanine, or phenylalanine, thereby conferring resistance to sulfonylurea, imidazolinone, and aryloxyphenoxypropionate herbicides (Table 1). In 2022, Han et al. [44] developed a highly efficient wheat ABE (WhieABE) based on the hyperactive adenine deaminase TadA8e. WhieABE was used to edit wheat tubulin alleles located on chromosomes 1A, 1B, 1D, 4A, and 4D, introducing A-to-G edits that caused Met-to-Thr substitutions in wheat tubulin and enhanced resistance to dinitroaniline herbicides (Table 1).

### 3.3. Application of Base Editing Technology in Other Crops

Beyond the major cereal crops, base editing technology has also been applied in other fields and horticultural crops. In 2019, Veillet et al. [45] used a CBE system to edit the *StGBSS1* gene in tetraploid potato, introducing base substitutions within the KTGGL catalytic domain, successfully applying base editing to a polyploid crop and creating potato mutants with impaired amylose biosynthesis across four alleles (Table 1). In 2018, Kang et al. [46] applied an ABE system to edit the *FT* and *PDS3* genes in Arabidopsis and Brassica napus protoplasts, generating either single amino acid substitutions in FT or aberrant splicing in PDS3 transcripts, resulting in transgenic plants with delayed flowering or albino phenotypes (Table 1). In 2020, Wu et al. [47] applied CBE to edit *BnALS1* and *BnALS3* genes in rapeseed, introducing C-to-T substitutions at codon P197, thereby creating transgene-free homozygous rapeseed mutants resistant to bensulfuron (Table 1). In the same year, Qin et al. [48] developed a novel cotton base-editing system, GhBE3, based on CBE, and edited the *GhCLA* and *GhPEBP* genes, achieving efficiencies of 26.67% and 57.78%, respectively (Table 1). In 2022, Wang et al. [49] applied ABE for the first time in allotetraploid cotton, achieving A-to-G editing efficiencies up to 64% at genomic target sites. They further developed a new editor, GhABE7.10dCpf1 (7.10TadA+dCpf1), which recognizes T-rich PAMs and successfully edited the *GhPEBP* gene to introduce amino acid substitutions that generated a compact plant architecture, serving as a prototype for mechanized cotton harvesting (Table 1). In 2020, Li et al. [50] used CBE to edit two non-allelic acetolactate synthase genes in maize, *ZmALS1* and *ZmALS2*, with in vivo C-to-T efficiencies up to 13.9%, successfully generating transgene-free homozygous *ZmALS1* mutants or double mutants conferring resistance to chlorsulfuron (Table 1). More recently, Fu et al. (2025) [51] tested optimized cytosine and adenine base editors (evoAPOBEC1, evoFERNY, evoCDA1, TadA8.20, TadA8e) in maize, editing *ZmACC1* and *ZmACC2* to generate herbicide-resistant materials. Among them, *ZmACC2* single mutants showed greater resistance than *ZmACC1* single mutants, while homozygous double mutants appeared to be lethal (Table 1). In 2017, Shimatani et al. [52] applied a codon-optimized PmCDA1 system in Arabidopsis to edit endogenous *DELLA* and *ETR1* genes in tomato, obtaining homozygous DELLA mutants with reduced serrated leaflet formation (Table 1). In 2019, Veillet et al. [53] used a CBE system to edit *SlALS* in tomato and *StALS* in potato, successfully generating chlorsulfuron-resistant plants, with tomato editing efficiencies reaching as high as 71% (Table 1). In 2018, Tian et al. [54] applied a modified BE3 system in watermelon to edit the *ALS* gene, successfully creating herbicide-resistant germplasm resources (Table 1).

## 4. Prospects of Base Editing Technology in Crop Applications

### 4.1. Limitations of Base Editing Technology

Compared with traditional genome editing technologies such as CRISPR/Cas9, base editing technology demonstrates significant advantages, including high target specificity, precise and efficient editing, and a low frequency of off-target effects [55]. In recent years, with the continuous optimization of research systems, this technology has achieved remarkable progress, but it still has certain limitations. For example, in practical applications, it is restricted by multiple factors such as editing efficiency, PAM recognition range, editing window width, types of base conversion, and off-target effects. Systematic optimization of these factors remains the key to advancing the application of base editing technology in crop breeding.

#### 4.1.1. Factors Affecting Editing Efficiency

Editing efficiency has always been one of the focal points of genome editing technology, and many factors influence the efficiency of base editing. The main aspects include the following: First, the efficiency differs among various types of base editors [51]. CBE and ABE were the earliest developed, and after continuous optimization, their editing efficiency has become relatively substantial, reaching as high as 100% at some sites, whereas GBE generally shows lower efficiency. Therefore, optimizing existing base editors and developing novel base editors for better application in plants is still necessary. Second, the design of sgRNA and the choice of target sites play important roles: GC content, secondary structure, and complementarity to the target site significantly affect efficiency. It is noteworthy that in certain genes or target sites, base editing can only achieve extremely low efficiency or may even fail entirely [56]. Third, the expression levels of Cas proteins and sgRNAs also influence editing efficiency. Generally, the higher the expression levels of Cas proteins and sgRNAs, the higher the editing efficiency. Selecting and optimizing strong promoters to enhance their expression can improve efficiency [57]. At the same time, codon optimization of Cas proteins according to codon usage bias in different plant species can also increase protein expression levels and thereby improve editing efficiency [58]. Fourth, nuclear localization signals (NLS) also affect base editing efficiency. For instance, from CBE4 to CBEmax, the adoption of bipartite NLS (bis-bpNLS) significantly enhanced editing efficiency [12]. Fifth, plant species themselves also affect editing efficiency [59]. In rice, relatively high editing efficiency has already been achieved [33], whereas in crops such as wheat, the editing efficiency remains relatively low [44]. These differences may be attributed to factors such as genomic complexity of different crops (e.g., large genome sizes and redundant homologous genes in polyploid species), challenges in transformation, and variations in expression level and stability. Editing efficiency can be substantially improved through strategies such as optimizing delivery methods, employing strong promoters to enhance editor expression, selecting high-efficiency Cas variants, designing sgRNAs with predictive algorithms for improved editability, and utilizing more active deaminases in specific plant contexts. In addition to the multiple factors affecting editing efficiency mentioned above, the application of base editors is also severely constrained by the strict PAM recognition requirements, representing a critical bottleneck in current technological development.

#### 4.1.2. Limitations of PAM Regions

Base mutations are not effective at just any position; usually, mutations only have meaningful effects at specific sites. However, such specific sites do not always contain the essential PAM sequence (NGG) required by SpCas9, which prevents targeted base editing from occurring. Therefore, expanding the PAM recognition scope is particularly important for base editing technology. Many researchers have recently modified SpCas9 to expand the number of recognizable PAM sites. In 2018, David R. Liu’s team employed phage-assisted continuous evolution (PACE) to create the xCas9 3.7 variant, which can recognize NGG, NG, GAA, and GAT PAMs [60]. In the same year, Nishimasu et al. developed the SpCas9-NG variant with stronger activity, which extended PAM recognition to NG sequences [28]. In 2020, Zhang et al. used the tRNA-esgRNA system in rice to successfully develop an efficient cytosine base editor, xCas9n-epBE, capable of achieving efficient C-to-T conversions at sites with expanded GA PAMs and even relaxed NG PAMs [61]. In 2019, Wang et al. fused the SpCas9-NG variant with optimized base editors such as Anc689BE4max, successfully applying it to edit genes such as acetolactate synthase (*ALS*) and 5-enolpyruvylshikimate-3-phosphate synthase (*EPSPS*) in rice at noncanonical NG PAM sites (e.g., AGC, TGA), with editing efficiencies ranging from 17.2% to 57.1%, thereby expanding the target range and obtaining herbicide-resistant mutants [62]. Walton et al. (2020) designed the SpG variant by introducing point mutations (D1136L, S1218W, G1219K, E1335Q, R1337Q, and T1R) into SpCas9-VRQR, thereby expanding PAM recognition to NGN. In human cells, SpG achieved a 51.2% editing efficiency at NGG sites. On this basis, they further introduced five additional mutations (A61R, L1111R, N1317R, A1322R, and R1333P) to develop the SpRY variant, which is currently the Cas9 variant most compatible with PAM sequences and can target nearly all PAMs [63]. Similarly, Miller et al. (2020) employed phage-assisted noncontinuous evolution (PANCE) and three new PACE strategies to generate three new SpCas9 variants that can recognize non-G PAMs (NRNH, where R = A or G, and H = A, C, or T), specifically NRRH, NRTH, and NRCH PAMs [64]. Zhang et al. (2021) optimized SpRY with plant-preferred codons (SpRYn) and constructed CBE editing systems such as SpRYn-CBE (NCN, NTH, NAG, and NAC), SpNRRHn-CBE (NAA), and SpNRTHn-CBE (NAT). In rice, these systems expanded the targeting scope of CBE nearly to PAM-free levels, except for NTG. As for ABE, SpRY expanded PAM compatibility in rice to NAB (B = C, T, or G), NCR (R = A or G), NTK (K = T or G), and NGV (V = A, C, or G) PAMs [65]. In addition, Cas12 (also known as Cpf1) is another Class II CRISPR nuclease widely applied in genome editing after Cas9. Cas12 proteins can recognize T-rich PAM sequences (TTTV), complementing Cas9’s preference for G-rich PAMs, though reports on the use of Cas12 in base editors remain relatively limited [66,67,68,69]. Li et al. (2018) developed the dCpf1-eBE system based on Cpf1, which recognizes TTTV PAMs and enables targeted base editing with extremely low levels of indels [67]. Kleinstiver et al. engineered AsCas12a, and the enAsCas12a (E174R/S542R/K548R) variant could recognize TTYN (Y = T or C), VTTV (V = A, C, or G), and TRTV (R = A or G) PAMs, exhibiting twice the editing activity of wild-type AsCas12a at TTTV PAM sites [68]. In 2023, Gaillochet et al. reported that wild-type LbCas12a-ABE showed low or no activity in plants. Using the ITER platform, they optimized LbCas12a-ABE and achieved up to 55% editing efficiency in stable wheat lines, providing valuable references for Cas12-based base editing applications in plants [69]. Cheng et al. (2023) fused highly efficient deaminases (hA3A-Y130F or ecTadA8e) with the optimized dLbCas12a-D156R protein to successfully develop Cas12a-based base editors that achieved nearly 100% efficient base editing (C-to-T or A-to-G) in rice at T-rich PAM sites (TTTV), thus broadening the application scope of plant genome editing [70]. In 2023, Lv et al. applied the CRISPR/Cas12i3 system to rice, taking advantage of its TTN PAM recognition, and developed the iMAGE system (interspaced array of direct repeats for genome editing), which achieved editing efficiencies of up to 47.3% and was successfully used for gene knockout and chromosomal structural variation in rice [71]. Despite the increasing number of PAM options, there are still many blind spots within the vast plant genome sequences. Beyond the constraints imposed by PAM sequences, the breadth of the editing window directly determines the flexibility and precision of base editing, posing another technical challenge that requires urgent resolution.

#### 4.1.3. Limitations of the Editing Window

Currently, the mainstream base editing systems, CBE and ABE, possess editing window widths of only 3–10 nucleotides (typically concentrated in the 5–7 nt range). This narrow window means that a single editing event can only cover substitutions for 2–3 amino acids and is prone to producing non-target “bystander editing,” where other C or A bases within the window are also edited [72,73]. Researchers have attempted to reduce bystander editing through engineered modifications of deaminases. For example, Jeong et al. [74] introduced a D108Q mutation into the catalytic domain TadA7.10 of ABE, selectively suppressing cytidine deamination activity while retaining adenine editing efficiency, thereby significantly reducing bystander cytidine editing in ABEs. Chen et al. [75] introduced N108Q and L145T double mutations into ABE8e to construct ABE9, which narrowed the editing window to 1–2 nt, directly avoiding bystander editing of adjacent adenines. Hao et al. [76] adopted a different approach, leveraging the smaller size of Cas12b compared with Cas9 to reduce steric hindrance when fused with deaminases. By engineering Cas12b, they developed a novel base editor with a significantly expanded editing window (19–43 nt) and optimized efficiency, while reducing bystander editing through specific strategies. Recently, Tang Weixin’s team [77] applied directed evolution to engineer the substrate recognition domain of TadA deaminase, enabling specific editing of 16 NCN sequences. Although the editing window was not significantly expanded, precision was greatly enhanced and bystander editing—especially at consecutive cytosine sites—was reduced. The team led by Samuele G. Marro engineered a TadA variant (TadA-NW1) by introducing mutations into the substrate-binding pocket of TadA-8e, guided by structural mimicry of the RNA-binding domain of human Pumilio1 protein. When fused with Cas9, this variant narrows the editing window from 10 bp to 4 bp, maintaining high on-target editing efficiency while substantially reducing off-target effects [78]. The central challenge of base editing windows lies in the difficulty of simultaneously optimizing editing width, efficiency, and specificity. Strategies to achieve a balance that maximizes benefits still require further research. Apart from the limitation imposed by the editing window width, the narrow spectrum of permissible base substitutions significantly restricts the applications of base editors, driving the development of novel editing tools.

#### 4.1.4. Limited Types of Base Substitutions

Although base editing systems have been continuously developed and expanded by researchers, the range of achievable base substitutions remains limited. The main systems include: the cytosine base editor (CBE) for C-to-T substitution, the adenine base editor (ABE) for A-to-G substitution, the glycosylase base editor (GBE) for C-to-G transversion (achieved by fusing UNG with CBE), and more recently, the AYBE system that fuses an optimized human MPG protein to the C-terminus of ABE8e for A-to-Y (Y = C or T) transversion [79]. In rice, the AKBE system has been developed for A-to-K (K = G or T) transversions [80,81], while the gTBE system, which fuses nCas9 with an engineered human uracil DNA glycosylase (UNG) variant, enables T-to-S (S = C or G) substitutions [82]. In addition, in 2019 Anzalone and colleagues developed the prime editing system (PE) [83], which consists of a reverse transcriptase (RT), nCas9 protein, and a prime editing guide RNA (pegRNA). This system is capable of introducing small, targeted insertions, deletions, and up to twelve types of base substitutions (C-to-T, G-to-A, A-to-G, T-to-C, C-to-A, C-to-G, G-to-C, G-to-T, A-to-C, A-to-T, T-to-A, T-to-G) in human cells. Mechanistically, under pegRNA guidance, nCas9 cleaves the DNA strand containing the PAM site, after which the broken DNA strand hybridizes with the 3′ extension of the pegRNA. Reverse transcription is then initiated along the RT template sequence to generate a DNA strand carrying the intended mutation, which is integrated into the genome following DNA ligation and repair, thus enabling precise editing. Compared with conventional base editing systems, PE is more precise and versatile, effectively overcoming the limitation of restricted base substitution types, and is particularly suitable for complex genome editing. In 2020, research groups led by Jiankang Zhu, Caixia Gao, and Lanqin Xia first applied PE to plants, but the efficiency remained unstable and relatively low, with a maximum of only 21.8% [84,85,86]. Jiang et al. (2022) optimized PE and successfully generated homozygous EPSPS-TAP-IVS mutant rice germplasm with glyphosate resistance, achieving a maximum editing efficiency of 6.1% in transgenic plants [87]. Qiao et al. (2023) applied an optimized PE system in maize to create germplasm resistant to glyphosate (EPSPS-TAP-IVS mutation), ACCase inhibitors (ZmACC1-W2284G mutation), and ALS inhibitors (ZmALS-W542L/S621I mutations), reaching maximum editing efficiencies of 12.0% (homozygous) and 17.8% (heterozygous) [88]. Gupta et al. (2023) utilized a highly efficient PE system in rice to create dominant bacterial blight-resistant germplasm by inserting a 30-bp effector-binding element (EBE) into the promoter of *Xa23*, and recessive resistant germplasm by introducing a V39E point mutation into the coding region of *TFIIAγ5*, with editing efficiencies of 73.6% and 88.5%, respectively [89]. Ni et al. (2023) applied an optimized PE system in hexaploid wheat and successfully generated transgenic plants simultaneously edited at eight endogenous genes (*TaWTK3*, *TaALS-T2*, *TaACC-T2*, *TaSBEIIa*, *TaLOX2*, *TaDME*, *TaGW2*, and *TaGASR7*), achieving editing efficiencies ranging from 19.6% to 86.3%, with the highest efficiency at the *TaGASR7* locus (86.3%) [90]. Although prime editing holds great promise, it still faces significant challenges, including relatively low editing efficiency, the complexity of pegRNA design (which must contain both targeting and editing template sequences), and safety concerns associated with introducing larger sequence changes, which hinder large-scale applications [83]. Therefore, compared with PE, base editing offers the advantages of higher efficiency and fewer base alterations, making it the preferred choice for applicable edits, while PE is mainly suitable for edits involving multiple base changes or those not achievable by base editors. In addition to the aforementioned technical limitations, off-target effects during base editing represent a major concern for the safety of practical applications, particularly requiring stringent control in large-scale crop breeding programs.

#### 4.1.5. Off-Target Effects

Off-target activity is a common challenge across all genome editing platforms. Compared with conventional CRISPR editing systems, base editors utilize Cas9 variants that do not introduce double-strand breaks (DSBs), significantly reducing—but not eliminating—the occurrence of off-target events [91,92]. One source of off-target activity is the nonspecific binding of sgRNA to homologous sequences in the genome, leading to unpredictable edits. Another source arises from the deaminase, the catalytic core of base editors, whose activity and specificity influence off-target profiles. Deaminases may dissociate from Cas proteins and independently bind DNA, resulting in unintended DNA edits, or they may act on RNA, generating RNA off-target effects. Engineering deaminases has proven effective in reducing off-target events. Numerous studies show that modifications to APOBEC, such as R33A or R33A/K34A mutations, or using eA3A as the deaminase, can lower both DNA and RNA off-target effects [93]. Hu et al. (2025) embedded the deaminase within a circularly permuted Cas9(D10A) protein to construct a new base editor architecture, QBEmax, which not only achieved efficient targeted editing but also minimized indel formation, exhibited high product purity, and substantially reduced DNA off-target activity [94]. Although off-target effects are less concerning in crop improvement compared to therapeutic applications in mammalian cells, further research is still needed to minimize unintended edits in agricultural breeding.

### 4.2. Summary and Outlook

As the third generation of genome editing technology, base editing (BE) integrates deaminases with the CRISPR/Cas system to achieve targeted modification of DNA or RNA bases. With advantages such as high precision, efficiency, and the ability to operate without introducing double-strand breaks (DSBs), BE has shown great promise in crop genetic improvement. By precisely modifying herbicide-resistance genes (e.g., *OsALS*), disease/stress-resistance genes, and quality-related genes, researchers have successfully generated novel germplasm with enhanced herbicide resistance, stronger stress tolerance, and improved quality. Although prime editing (PE) has advanced rapidly and allows for arbitrary base conversions and more complex genome rewriting, it is important to note that regulatory policies in countries such as the United States, Japan, Argentina, and China generally classify gene-edited plants lacking exogenous DNA—where only point mutations or small fragment deletions occur—as non-transgenic, or simplify their safety evaluation process [95]. This means that base editing, which only introduces point mutations, faces fewer regulatory restrictions and is more readily accepted and applied. In contrast, PE applications involving large-fragment insertions remain subject to stricter transgenic-related regulations. Therefore, base editing still holds significant practical value in plants. Its future development may focus on the following directions: (1) Optimization of editor performance: This involves further engineering of deaminases and Cas proteins through protein engineering and directed evolution to enhance efficiency, expand the editing window, and minimize bystander editing. (2) Expansion of PAM compatibility: This involves the development of Cas variants with relaxed PAM recognition to enlarge the editable genomic scope. (3) Application and diversification of editors: This involves promoting the application of existing editors such as GBE and AKBE, while developing novel base editors to enrich the spectrum of achievable base conversions. (4) Exploration of novel editing platforms: This involves establishing highly efficient Cas12-based base editing systems and other innovative approaches. (5) Integration with Artificial Intelligence (AI): In recent years, the field of AI has advanced rapidly, demonstrating revolutionary potential in areas such as medicine and agriculture. Deep integration of AI with base editing technology can accelerate its development toward greater speed and precision. For instance, Wei et al. recently employed the AI model ProMEP to perform a series of protein engineering efforts on Cas9, successfully designing a high-performance and highly versatile Cas9 variant (Cas9-AI-8.3). This variant enhances the efficiency of multiple mainstream base editors by an average of 2- to 3-fold [96]. First, AI enables precise navigation and target prediction by deeply mining multi-omics data to accurately identify key single-nucleotide variations associated with crop quality, yield, and disease resistance. Second, AI-driven protein design platforms such as AlphaFold [97] and RFdiffusion [98] can rapidly and accurately resolve the three-dimensional structures of base editors. These platforms facilitate de novo design or optimization of components such as deaminases, Cas proteins, and linkers, thereby improving the performance of base editors. Finally, AI models trained on vast amounts of on-target and off-target editing data can learn to identify genomic sequence features that may lead to unintended edits. This allows for the prediction of off-target risks before the deployment of base editors, enhancing their safety profile. In summary, base editing technology is steadily maturing, with broad application prospects in crop breeding. It is expected to provide powerful technological support for global food security and sustainable agricultural development.

## Figures and Tables

**Table 1:** Applications of Base Editing Systems in Various Crops.

Species	Base Editors	Target Gene	Editing Efficiency (%)	Transformation	Function	References
Rice	BE3	*OsSBEIIb*, *OsPDS*	20.00	*Agrobacterium*	Starch structure	[39]
	APOBEC1-XTEN-Cas9n	*NRT1.1B*, *SLR1*	2.70–13.30	*Agrobacterium*	Nitrogen absorption efficiency, Plant height, Lodging resistance	[40]
	PABE	*OsACC-T1*, *OsALS-T1*, *OsCDC48-T3*, *OsDEP1-T1*, *OsDEP1-T2*, *OsNRT1.1B-T1*	15.80–59.10	*Agrobacterium*	Herbicide resistance	[29]
	rBE9, rBE14	*OsALS1*	15.20–23.80	*Agrobacterium*, Bombardment	Herbicide resistance	[41]
	rBE14	*OsTubA2*	12.70	*Agrobacterium*	Herbicide resistance	[42]
	CGBE	*OsIPA1*, *OsbZIP5*, *OsSIR1*, *OsALS1*, *NRT1.18*	21.3	*Agrobacterium*	Herbicide resistance, Nitrogen utilization rate	[36]
Wheat	pnCas9-PBE	*TaALS*	2.50	Bombardment	Herbicide resistance	[43]
	WhieABE8e	tubulin genes	78.00	*Agrobacterium*	Herbicide resistance	[44]
Potato	CBE	*StGBSSI*	90.00	*Agrobacterium*	Amylose content	[45]
*Arabidopsis*, Rapeseed	pcABE7.10	*AtALS*, *BnPDS*	4.10–50.00	*Agrobacterium*	Late flowering, Whitening	[46]
	CBE	*BrALS1*	1.80	*Agrobacterium*	Herbicide resistance	[47]
Cotton	GhBE3	*GhCLA*, *GhPEBP*	37.32	*Agrobacterium*	Whitening	[48]
	GhABE7.10n	*GhPEBP*	64.00	*Agrobacterium*	Compact structure	[49]
Corn	CBE	*ZmALS1*, *ZmALS2*	13.90	*Agrobacterium*	Herbicide resistance	[50]
	evoCDA1, TadA8.20	*ZmACC1*, *ZmACC2*	79.00–100.00	*Agrobacterium*	Herbicide resistance	[51]
Tomatoes, Potatoes	Target-AID	*DELLA*, *ETR1*	18.30	*Agrobacterium*	Leaf morphology	[52]
	Target-AID	*SlALS*, *StALS*	70.00	*Agrobacterium*	Herbicide resistance	[53]
Watermelon	BE3	*ClALS*	23.00	*Agrobacterium*	Herbicide resistance	[54]

## References

[1] Durai S., Mani M., Kandavelou K., Wu J., Porteus M.H., Chandrasegaran S. (2005). Zinc finger nucleases: Custom-designed molecular scissors for genome engineering of plant and mammalian cells. Nucleic Acids Res..

[2] Joung J.K., Sander J.D. (2013). TALENs: A widely applicable technology for targeted genome editing. Nat. Rev. Mol. Cell Biol..

[3] Cong L., Ran F.A., Cox D., Lin S., Barretto R., Habib N., Hsu P.D., Wu X., Jiang W., Marraffini L.A. (2013). Multiplex genome engineering using CRISPR/Cas systems. Science.

[4] Lieber M.R. (2010). The mechanism of double-strand DNA break repair by the nonhomologous DNA end-joining pathway. Annu. Rev. Biochem..

[5] Zhao H.Y., Xu D.Y. (2021). Selection and regulation of DNA double-strand break repair pathways. Sci. Sin. Vitae.

[6] Henikoff S., Comai L. (2003). Single-nucleotide mutations for plant functional genomics. Annu. Rev. Plant Biol..

[7] Huq M.A., Akter S., Nou I.S., Kim H.T., Jung Y.J., Kang K.K. (2016). Identification of functional SNPs in genes and their effects on plant phenotypes. J. Plant Biotechnol..

[8] Miura K., Ikeda M., Matsubara A., Song X.J., Ito M., Asano K., Matsuoka M., Kitano H., Ashikari M. (2010). *OsSPL14* promotes panicle branching and higher grain productivity in rice. Nat. Genet..

[9] Su Z., Hao C., Wang L., Dong Y., Zhang X. (2011). Identification and development of a functional marker of *TaGW2* associated with grain weight in bread wheat (*Triticum aestivum* L.). Theor. Appl. Genet..

[10] Komor A.C., Kim Y.B., Packer M.S., Zuris J.A., Liu D.R. (2016). Programmable editing of a target base in genomic DNA without double-stranded DNA cleavage. Nature.

[11] Komor A.C., Zhao K.T., Packer M.S., Gaudelli N.M., Waterbury A.L., Koblan L.W., Kim Y.B., Badran A.H., Liu D.R. (2017). Improved base excision repair inhibition and bacteriophage Mu Gam protein yields C:G-to-T:A base editors with higher efficiency and product purity. Sci. Adv..

[12] Koblan L.W., Doman J.L., Wilson C., Levy J.M., Tay T., Newby G.A., Maianti J.P., Raguram A., Liu D.R. (2018). Improving cytidine and adenine base editors by expression optimization and ancestral reconstruction. Nat. Biotechnol..

[13] Gehrke J.M., Cervantes O., Clement M.K., Wu Y., Zeng J., Bauer D.E., Pinello L., Joung J.K. (2018). An APOBEC3A-Cas9 base editor with minimized bystander and off-target activities. Nat. Biotechnol..

[14] Thuronyi B.W., Koblan L.W., Levy J.M., Yeh W.H., Zheng C., Newby G.A., Wilson C., Bhaumik M., Shubina-Oleinik O., Holt J.R. (2019). Continuous evolution of base editors with expanded target compatibility and improved activity. Nat. Biotechnol..

[15] Chen L., Zhu B., Ru G., Meng H., Yan Y., Hong M., Zhang D., Luan C., Zhang S., Wu H. (2023). Re-engineering the adenine deaminase TadA-8e for efficient and specific CRISPR-based cytosine base editing. Nat. Biotechnol..

[16] Neugebauer M.E., Hsu A., Arbab M., Krasnow N.A., McElroy A.N., Pandey S., Doman J.L., Huang T.P., Raguram A., Banskota S. (2023). Evolution of an adenine base editor into a small, efficient cytosine base editor with low off-target activity. Nat. Biotechnol..

[17] Gaudelli N.M., Komor A.C., Rees H.A., Packer M.S., Badran A.H., Bryson D.I., Liu D.R. (2017). Programmable base editing of A•T to G•C in genomic DNA without DNA cleavage. Nature.

[18] Gaudelli N.M., Lam D.K., Rees H.A., Solá-Esteves N.M., Barrera L.A., Born D.A., Edwards A., Gehrke J.M., Lee S.J., Liquori A.J. (2020). Directed evolution of adenine base editors with increased activity and therapeutic application. Nat. Biotechnol..

[19] Richter M.F., Zhao K.T., Eton E., Lapinaite A., Newby G.A., Thuronyi B.W., Wilson C., Koblan L.W., Zeng J., Bauer D.E. (2020). Phage-assisted evolution of an adenine base editor with improved Cas domain compatibility and activity. Nat. Biotechnol..

[20] Zhao D., Li J., Li S., Xin X., Hu M., Price M.A., Rosser S.J., Bi C., Zhang X. (2021). Glycosylase base editors enable C-to-A and C-to-G base changes. Nat. Biotechnol..

[21] Kurt I.C., Zhou R., Iyer S., Garcia S.P., Miller B.R., Langner L.M., Grünewald J., Joung J.K. (2021). CRISPR C-to-G base editors for inducing targeted DNA transversions in human cells. Nat. Biotechnol..

[22] Sun N., Zhao D., Li S., Zhang Z., Bi C., Zhang X. (2022). Reconstructed glycosylase base editors GBE2.0 with enhanced C-to-G base editing efficiency and purity. Mol. Ther..

[23] Dong X., Yang C., Ma Z., Chen M., Zhang X., Bi C. (2022). Enhancing glycosylase base-editor activity by fusion to transactivation modules. Cell Rep..

[24] Chen L., Park J.E., Paa P., Rajakumar P.D., Prekop H.T., Chew Y.T., Manivannan S.N., Chew W.L. (2021). Programmable C:G to G:C genome editing with CRISPR-Cas9-directed base excision repair proteins. Nat. Commun..

[25] Zong Y., Wang Y., Li C., Zhang R., Chen K., Ran Y., Qiu J.L., Wang D., Gao C. (2017). Precise base editing in rice, wheat and maize with a Cas9-cytidine deaminase fusion. Nat. Biotechnol..

[26] Zong Y., Song Q., Li C., Jin S., Zhang D., Wang Y., Qiu J.L., Gao C. (2018). Efficient C-to-T base editing in plants using a fusion of nCas9 and human APOBEC3A. Nat. Biotechnol..

[27] Zeng D., Liu T., Tan J., Zhang Y., Zheng Z., Wang B., Zhou D., Xie X., Guo M., Liu Y.G. (2020). PhieCBEs: Plant High-Efficiency Cytidine Base Editors with Expanded Target Range. Mol. Plant.

[28] Nishimasu H., Shi X., Ishiguro S., Gao L., Hirano S., Okazaki S., Noda T., Abudayyeh O.O., Gootenberg J.S., Mori H. (2018). Engineered CRISPR-Cas9 nuclease with expanded targeting space. Science.

[29] Li C., Zong Y., Wang Y., Jin S., Zhang D., Song Q., Zhang R., Gao C. (2018). Expanded base editing in rice and wheat using a Cas9-adenosine deaminase fusion. Genome Biol..

[30] Yan F., Kuang Y., Ren B., Wang J., Zhang D., Lin H., Yang B., Zhou X., Zhou H. (2018). Highly Efficient A·T to G·C Base Editing by Cas9n-Guided tRNA Adenosine Deaminase in Rice. Mol. Plant.

[31] Wei C., Wang C., Jia M., Guo H.X., Luo P.Y., Wang M.G., Zhu J.K., Zhang H. (2021). Efficient generation of homozygous substitutions in rice in one generation utilizing an rABE8e base editor. J. Integr. Plant Biol..

[32] Yan D., Ren B., Liu L., Yan F., Li S., Wang G., Sun W., Zhou X., Zhou H. (2021). High-efficiency and multiplex adenine base editing in plants using new TadA variants. Mol. Plant.

[33] Tan J., Zeng D., Zhao Y., Wang Y., Liu T., Li S., Xue Y., Luo Y., Xie X., Chen L. (2022). PhieABEs: A PAM-less/free high-efficiency adenine base editor toolbox with wide target scope in plants. Plant Biotechnol. J..

[34] Zhang X., Chen L., Zhu B., Wang L., Chen C., Hong M., Huang Y., Li H., Han H., Cai B. (2020). Increasing the efficiency and targeting range of cytidine base editors through fusion of a single-stranded DNA-binding protein domain. Nat. Cell Biol..

[35] Sretenovic S., Liu S., Li G., Cheng Y., Fan T., Xu Y., Zhou J., Zheng X., Coleman G., Zhang Y. (2021). Exploring C-To-G Base Editing in Rice, Tomato, and Poplar. Front. Genome Ed..

[36] Tian Y., Shen R., Li Z., Yao Q., Zhang X., Zhong D., Tan X., Song M., Han H., Zhu J.K. (2022). Efficient C-to-G editing in rice using an optimized base editor. Plant Biotechnol. J..

[37] Fan T., Cheng Y., Wu Y., Liu S., Tang X., He Y., Liao S., Zheng X., Zhang T., Qi Y. (2024). High performance TadA-8e derived cytosine and dual base editors with undetectable off-target effects in plants. Nat. Commun..

[38] Jiang Y., Xiao Z., Luo Z., Zhou S., Tong C., Jin S., Liu X., Qin R., Xu R., Pan L. (2025). Improving plant C-to-G base editors with a cold-adapted glycosylase and TadA-8e variants. Trends Biotechnol..

[39] Li J., Sun Y., Du J., Zhao Y., Xia L. (2017). Generation of Targeted Point Mutations in Rice by a Modified CRISPR/Cas9 System. Mol. Plant.

[40] Lu Y., Zhu J.K. (2017). Precise Editing of a Target Base in the Rice Genome Using a Modified CRISPR/Cas9 System. Mol. Plant.

[41] Kuang Y., Li S., Ren B., Yan F., Spetz C., Li X., Zhou X., Zhou H. (2020). Base-Editing-Mediated Artificial Evolution of *OsALS1* In Planta to Develop Novel Herbicide-Tolerant Rice Germplasms. Mol. Plant.

[42] Liu L., Kuang Y., Yan F., Li S., Ren B., Gosavi G., Spetz C., Li X., Wang X., Zhou X. (2021). Developing a novel artificial rice germplasm for dinitroaniline herbicide resistance by base editing of *OsTubA2*. Plant Biotechnol. J..

[43] Zhang R., Liu J., Chai Z., Chen S., Bai Y., Zong Y., Chen K., Li J., Jiang L., Gao C. (2019). Generation of herbicide tolerance traits and a new selectable marker in wheat using base editing. Nat. Plants.

[44] Han H., Wu Z., Zheng L., Han J., Zhang Y., Li J., Wang P. (2022). Generation of a high-efficiency adenine base editor with TadA8e for developing wheat dinitroaniline-resistant germplasm. Crop J..

[45] Veillet F., Chauvin L., Kermarrec M.P., Sevestre F., Merrer M., Terret Z., Szydlowski N., Devaux P., Gallois J.L., Chauvin J.E. (2019). The Solanum tuberosum GBSSI gene: A target for assessing gene and base editing in tetraploid potato. Plant Cell Rep..

[46] Kang B.C., Yun J.Y., Kim S.T., Shin Y., Ryu J., Choi M., Woo J.W., Kim J.S. (2018). Precision genome engineering through adenine base editing in plants. Nat. Plants.

[47] Wu J., Chen C., Xian G., Liu D., Lin L., Yin S., Sun Q., Fang Y., Zhang H., Wang Y. (2020). Engineering herbicide-resistant oilseed rape by CRISPR/Cas9-mediated cytosine base-editing. Plant Biotechnol. J..

[48] Qin L., Li J., Wang Q., Xu Z., Sun L., Alariqi M., Manghwar H., Wang G., Li B., Ding X. (2020). High-efficient and precise base editing of C•G to T•A in the allotetraploid cotton (*Gossypium hirsutum*) genome using a modified CRISPR/Cas9 system. Plant Biotechnol. J..

[49] Wang G., Xu Z., Wang F., Huang Y., Xin Y., Liang S., Li B., Si H., Sun L., Wang Q. (2022). Development of an efficient and precise adenine base editor (ABE) with expanded target range in allotetraploid cotton (*Gossypium hirsutum*). BMC Biol..

[50] Li Y., Zhu J., Wu H., Liu C., Huang C., Lan J., Xie C. (2020). Precise base editing of non-allelic acetolactate synthase genes confers sulfonylurea herbicide resistance in maize. Crop J..

[51] Fu X., Wang N., Li L., Qiao D., Qi X., Liu C., Gao Z., Xie C., Zhu J. (2025). Development of cytosine and adenine base editors for maize precision breeding. J. Integr. Plant Biol..

[52] Shimatani Z., Kashojiya S., Takayama M., Terada R., Arazoe T., Ishii H., Teramura H., Yamamoto T., Komatsu H., Miura K. (2017). Targeted base editing in rice and tomato using a CRISPR-Cas9 cytidine deaminase fusion. Nat. Biotechnol..

[53] Veillet F., Perrot L., Chauvin L., Kermarrec M.P., Guyon-Debast A., Chauvin J.E., Nogué F., Mazier M. (2019). Transgene-Free Genome Editing in Tomato and Potato Plants Using Agrobacterium-Mediated Delivery of a CRISPR/Cas9 Cytidine Base Editor. Int. J. Mol. Sci..

[54] Tian S., Jiang L., Cui X., Zhang J., Guo S., Li M., Zhang H., Ren Y., Gong G., Zong M. (2018). Engineering herbicide-resistant watermelon variety through CRISPR/Cas9-mediated base-editing. Plant Cell Rep..

[55] Xu Q., Yang S., Hao H., Du W., Pang Y., Zhao S., Zhao X. (2021). Research Progress on the Application of Single Base Editing Technology. China Anim. Husb. Vet. Med..

[56] Hua K., Han P., Zhu J.K. (2022). Improvement of base editors and prime editors advances precision genome engineering in plants. Plant Physiol..

[57] Liu H., Wang K., Jia Z., Gong Q., Lin Z., Du L., Pei X., Ye X. (2020). Efficient induction of haploid plants in wheat by editing of *TaMTL* using an optimized Agrobacterium-mediated CRISPR system. J. Exp. Bot..

[58] Nanasato Y., Kawabe H., Ueno S., Konagaya K.I., Endo M., Taniguchi T. (2024). Improvement of genome editing efficiency by Cas9 codon optimization in Japanese cedar (*Cryptomeria japonica* D. Don). Plant Biotechnol..

[59] Vats S., Kumawat S., Kumar V., Patil G.B., Joshi T., Sonah H., Sharma T.R., Deshmukh R. (2019). Genome Editing in Plants: Exploration of Technological Advancements and Challenges. Cells.

[60] Hu J.H., Miller S.M., Geurts M.H., Tang W., Chen L., Sun N., Zeina C.M., Gao X., Rees H.A., Lin Z. (2018). Evolved Cas9 variants with broad PAM compatibility and high DNA specificity. Nature.

[61] Zhang C., Xu W., Wang F., Kang G., Yuan S., Lv X., Li L., Liu Y., Yang J. (2020). Expanding the base editing scope to GA and relaxed NG PAM sites by improved xCas9 system. Plant Biotechnol. J..

[62] Wang M., Wang Z., Mao Y., Lu Y., Yang R., Tao X., Zhu J.K. (2019). Optimizing base editors for improved efficiency and expanded editing scope in rice. Plant Biotechnol. J..

[63] Walton R.T., Christie K.A., Whittaker M.N., Kleinstiver B.P. (2020). Unconstrained genome targeting with near-PAMless engineered CRISPR-Cas9 variants. Science.

[64] Miller S.M., Wang T., Randolph P.B., Arbab M., Shen M.W., Huang T.P., Matuszek Z., Newby G.A., Rees H.A., Liu D.R. (2020). Continuous evolution of SpCas9 variants compatible with non-G PAMs. Nat. Biotechnol..

[65] Zhang C., Wang Y., Wang F., Zhao S., Song J., Feng F., Zhao J., Yang J. (2021). Expanding base editing scope to near-PAMless with engineered CRISPR/Cas9 variants in plants. Mol. Plant.

[66] Wang X., Ding C., Yu W., Wang Y., He S., Yang B., Xiong Y.C., Wei J., Li J., Liang J. (2020). Cas12a Base Editors Induce Efficient and Specific Editing with Low DNA Damage Response. Cell Rep..

[67] Li X., Wang Y., Liu Y., Yang B., Wang X., Wei J., Lu Z., Zhang Y., Wu J., Huang X. (2018). Base editing with a Cpf1-cytidine deaminase fusion. Nat. Biotechnol..

[68] Kleinstiver B.P., Sousa A.A., Walton R.T., Tak Y.E., Hsu J.Y., Clement K., Welch M.M., Horng J.E., Malagon-Lopez J., Scarfò I. (2019). Engineered CRISPR-Cas12a variants with increased activities and improved targeting ranges for gene, epigenetic and base editing. Nat. Biotechnol..

[69] Gaillochet C., Peña Fernández A., Goossens V., D’Halluin K., Drozdzecki A., Shafie M., Van Duyse J., Van Isterdael G., Gonzalez C., Vermeersch M. (2023). Systematic optimization of Cas12a base editors in wheat and maize using the ITER platform. Genome Biol..

[70] Cheng Y., Zhang Y., Li G., Fang H., Sretenovic S., Fan A., Li J., Xu J., Que Q., Qi Y. (2023). CRISPR-Cas12a base editors confer efficient multiplexed genome editing in rice. Plant Commun..

[71] Lv P., Su F., Chen F., Yan C., Xia D., Sun H., Li S., Duan Z., Ma C., Zhang H. (2024). Genome editing in rice using CRISPR/Cas12i3. Plant Biotechnol. J..

[72] Rees H.A., Liu D.R. (2018). Base editing: Precision chemistry on the genome and transcriptome of living cells. Nat. Rev. Genet..

[73] Porto E.M., Komor A.C., Slaymaker I.M., Yeo G.W. (2020). Base editing: Advances and therapeutic opportunities. Nat. Rev. Drug Discov..

[74] Jeong Y.K., Lee S., Hwang G.H., Hong S.A., Park S.E., Kim J.S., Woo J.S., Bae S. (2021). Adenine base editor engineering reduces editing of bystander cytosines. Nat. Biotechnol..

[75] Chen L., Zhang S., Xue N., Hong M., Zhang X., Zhang D., Yang J., Bai S., Huang Y., Meng H. (2023). Engineering a precise adenine base editor with minimal bystander editing. Nat. Chem. Biol..

[76] Hao W., Cui W., Liu Z., Suo F., Wu Y., Han L., Zhou Z. (2024). A New-Generation Base Editor with an Expanded Editing Window for Microbial Cell Evolution In Vivo Based on CRISPR—Cas12b Engineering. Adv. Sci..

[77] Wu Y., Xiao Y.L., Tang W. (2025). High-precision cytosine base editors by evolving nucleic-acid-recognition hotspots in deaminase. Nat. Biotechnol..

[78] Valdez I., O’Connor I., Patel D., Gierer K., Harrington J., Ellis E., Caponetti S.A., Sebra R.P., Valley H.C., Coote K. (2025). A streamlined base editor engineering strategy to reduce bystander editing. Nat. Commun..

[79] Tong H., Wang X., Liu Y., Liu N., Li Y., Luo J., Ma Q., Wu D., Li J., Xu C. (2023). Programmable A-to-Y base editing by fusing an adenine base editor with an N-methylpurine DNA glycosylase. Nat. Biotechnol..

[80] Li Y., Li S., Li C., Zhang C., Yan L., Li J., He Y., Guo Y., Lin Y., Zhang Y. (2023). Engineering a plant A-to-K base editor with improved performance by fusion with a transactivation module. Plant Commun..

[81] Wu X., Ren B., Liu L., Qiu S., Li X., Li P., Yan F., Lin H., Zhou X., Zhang D. (2023). Adenine base editor incorporating the N-methylpurine DNA glycosylase MPGv3 enables efficient A-to-K base editing in rice. Plant Commun..

[82] Tong H., Wang H., Wang X., Liu N., Li G., Wu D., Li Y., Jin M., Li H., Wei Y. (2024). Development of deaminase-free T-to-S base editor and C-to-G base editor by engineered human uracil DNA glycosylase. Nat. Commun..

[83] Anzalone A.V., Randolph P.B., Davis J.R., Sousa A.A., Koblan L.W., Levy J.M., Chen P.J., Wilson C., Newby G.A., Raguram A. (2019). Search-and-replace genome editing without double-strand breaks or donor DNA. Nature.

[84] Lin Q., Zong Y., Xue C., Wang S., Jin S., Zhu Z., Wang Y., Anzalone A.V., Raguram A., Doman J.L. (2020). Prime genome editing in rice and wheat. Nat. Biotechnol..

[85] Hua K., Jiang Y., Tao X., Zhu J.K. (2020). Precision genome engineering in rice using prime editing system. Plant Biotechnol. J..

[86] Li H., Li J., Chen J., Yan L., Xia L. (2020). Precise Modifications of Both Exogenous and Endogenous Genes in Rice by Prime Editing. Mol. Plant.

[87] Jiang Y., Chai Y., Qiao D., Wang J., Xin C., Sun W., Cao Z., Zhang Y., Zhou Y., Wang X.C. (2022). Optimized prime editing efficiently generates glyphosate-resistant rice plants carrying homozygous TAP-IVS mutation in EPSPS. Mol. Plant.

[88] Qiao D., Wang J., Lu M.H., Xin C., Chai Y., Jiang Y., Sun W., Cao Z., Guo S., Wang X.C. (2023). Optimized prime editing efficiently generates heritable mutations in maize. J. Integr. Plant Biol..

[89] Gupta A., Liu B., Chen Q.J., Yang B. (2023). High-efficiency prime editing enables new strategies for broad-spectrum resistance to bacterial blight of rice. Plant Biotechnol. J..

[90] Ni P., Zhao Y., Zhou X., Liu Z., Huang Z., Ni Z., Sun Q., Zong Y. (2023). Efficient and versatile multiplex prime editing in hexaploid wheat. Genome Biol..

[91] Jin S., Zong Y., Gao Q., Zhu Z., Wang Y., Qin P., Liang C., Wang D., Qiu J.L., Zhang F. (2019). Cytosine, but not adenine, base editors induce genome-wide off-target mutations in rice. Science.

[92] Zhou C., Sun Y., Yan R., Liu Y., Zuo E., Gu C., Han L., Wei Y., Hu X., Zeng R. (2019). Off-target RNA mutation induced by DNA base editing and its elimination by mutagenesis. Nature.

[93] Slesarenko Y.S., Lavrov A.V., Smirnikhina S.A. (2022). Off-target effects of base editors: What we know and how we can reduce it. Curr. Genet..

[94] Hu J., Guo M., Gao Q., Jia H., He M., Wang Z., Guo L., Liu G., Gao Q., Zhao K.T. (2025). QBEmax is a sequence-permuted and internally protected base editor. Nat. Biotechnol..

[95] Yang F., Zheng K., Yao Y. (2024). China’s regulatory change toward genome-edited crops. Trends Biotechnol..

[96] Wei D., Cheng P., Song Z., Liu Y., Xu X., Huang X., Wang X., Zhang Y., Shu W., Wei Y. (2025). AI-guided Cas9 engineering provides an effective strategy to enhance base editing. Mol. Syst. Biol..

[97] Jumper J., Evans R., Pritzel A., Green T., Figurnov M., Ronneberger O., Tunyasuvunakool K., Bates R., Žídek A., Potapenko A. (2021). Highly accurate protein structure prediction with AlphaFold. Nature.

[98] Watson J.L., Juergens D., Bennett N.R., Trippe B.L., Yim J., Eisenach H.E., Ahern W., Borst A.J., Ragotte R.J., Milles L.F. (2023). De novo design of protein structure and function with RFdiffusion. Nature.

